# Giant Scrotal Lymphoedema: A Case Series

**DOI:** 10.7759/cureus.48248

**Published:** 2023-11-04

**Authors:** Sanjeev Kumar, Atreyee Saha, Suresh Kumar, Pankaj Singh, Krishna Kant Singh

**Affiliations:** 1 General Surgery, King George's Medical University (KGMU), Lucknow, IND

**Keywords:** external genitalia, lymphatic filariasis, reconstructive surgery, debulking, scrotal lymphoedema

## Abstract

Giant scrotal lymphoedema is a rare condition caused by obstruction, aplasia, or hypoplasia of lymphatic vessels draining the external genitalia. While this condition can be congenital or acquired, the most common acquired cause of such lymphatic obstruction worldwide is lymphatic filariasis (LF).

We present a case series analysis of three patients of giant scrotal lymphoedema who were successfully treated for the condition in the Department of General Surgery, King George's Medical University (KGMU), Lucknow, with satisfactory post-operative recovery and minimal recurrence.

The first patient was a 45-year-old who had been living with the condition for 10 years, and the resected scrotal tissue weighed 35 kg. The second patient was a 45-year-old who was diagnosed with filariasis five years back before the condition set in, and the resected scrotal tissue weighed 32 kg. The third patient was a 22-year-old young man who had been diagnosed with the condition 10 years back, and the resected scrotal tissue weighed 25 kg.

Proper pre-operative evaluation was conducted in all three patients to establish the diagnosis of scrotal lymphoedema. The urethral catheterisation was conducted, which additionally helped to identify penile tissue intraoperatively. Careful exploration of scrotal tissue was conducted along with delineation of the penis from scrotal oedema. The surgical approach involved debulking scrotal lymphoedema with the reconstruction of scrotal skin while preserving penile tissue.

Patients with giant scrotal lymphoedema face the social stigma that creates physical disability. Hence, they end up seeking medical help from tertiary care centres after the disease has reached advanced stages and fibrosis has set in. However, single-stage debulking, along with reconstructive surgery (referred to as reduction scrotoplasty), yields promising results even in cases of very bulky scrotal lymphoedema, weighing up to 35 kg, as per our study.

## Introduction

Giant scrotal lymphoedema, also known as elephantiasis, can be caused by obstruction, aplasia or hypoplasia of lymphatic vessels. Though it can be caused by a neoplasm, radiotherapy or lymphadenectomy, most cases are usually caused by infection as a result of filariasis or lymphogranuloma venerum caused by Wuchereria bancrofti and certain serovars of Chlamydia trachomatis [1].

Giant scrotal lymphoedema is a disease of the tropics and sub-tropics [1]. However, giant variants are extremely rare even in endemic areas. Massive lymphoedema is usually caused secondary to obstruction or malformation of lymphatic channels draining the genital areas [2].

According to a recent classification, the main aetiological causes of lymphoedema can be primary (idiopathic) or secondary (infections such as lymphogranuloma venerum and filariasis, post-trauma/post-surgical, post-radiation therapy), resulting from disruption in lymphatic flow [3]. Among these causes, filariasis remains the leading cause of scrotal lymphoedema in developing countries worldwide. The condition causes severe degrees of physical limitations, making it difficult to maintain hygiene and social stigmata intervening with the daily physical activities of the patients [4]. This can cause immense physical as well as emotional and psychological disturbances to the patients. It can affect any age group, especially those living in areas endemic to lymphatic filariasis (LF). Early recognition remains the key approach to therapy as there is no definite cure for the condition [5]. The role of conservative treatment is limited to the early stages of the disease, but the effect is temporary and requires lifestyle modifications, limb care and strict follow-up. Anthelminthic drugs like diethyl carbamazepine and ivermectin have shown some role in the initial stages of the disease. Although they have a limited role in the cure of the disease, they can prevent upstaging of the disease when used during the initial stages. Therefore, the mainstay of treatment remains surgical resection of fibrotic tissues with reconstructive surgery of external genitalia. We present a case series analysis of three patients with giant scrotal lymphoedema, which were presented in the Department of General Surgery, King George's Medical University (KGMU), Lucknow.

## Case presentation

Case 1

A 45-year-old man presented with a chief complaint of a gradually enlarging swelling of the bilateral scrotum, accompanied by a repeated history suggestive of scrotal lymphangitis for the past 10 years. There was no history of any radiation exposure or trauma. The patient also revealed suffering from chronic depression and an inability to perform his daily activities due to this condition. A careful clinical examination revealed a large grade IV scrotal swelling of about 78 cm x 64 cm x 54 cm, with the penis buried inside a pit in the scrotum. There were multiple pits and fissures all over the scrotal skin. Difficulty in voiding urine and urinary soiling of scrotal skin led to associated multiple scrotal ulcers. Scrotal skin was pitted and fibrotic. Testis could not be palpated. Routine blood investigations were normal, and the imaging study like high-resolution ultrasonography excluded any abnormality in the inguinal region or lower abdomen.

**Figure 1:**
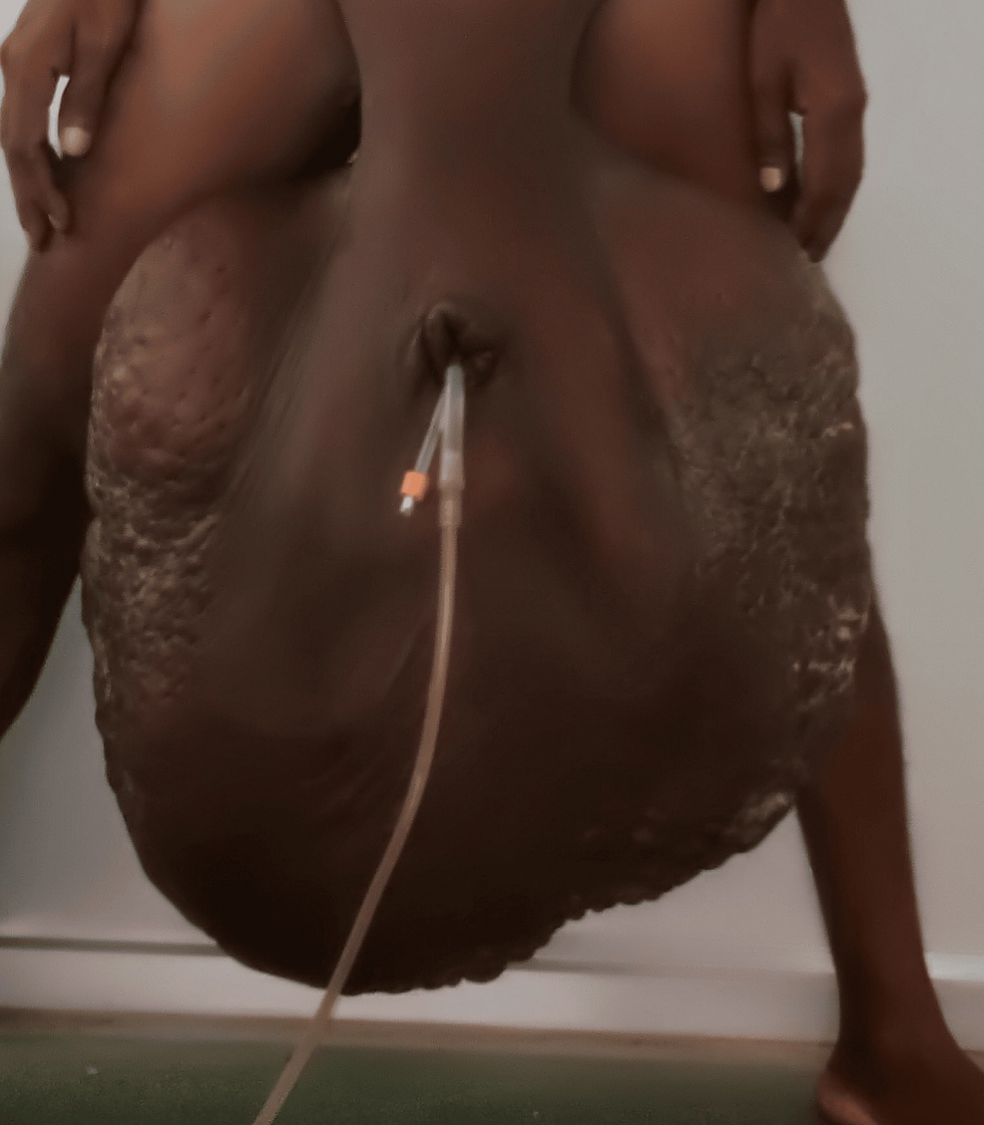
Pre-operative scrotum: note the hugely enlarged size with fibrotic tissue and the presence of fissures.

**Figure 2:**
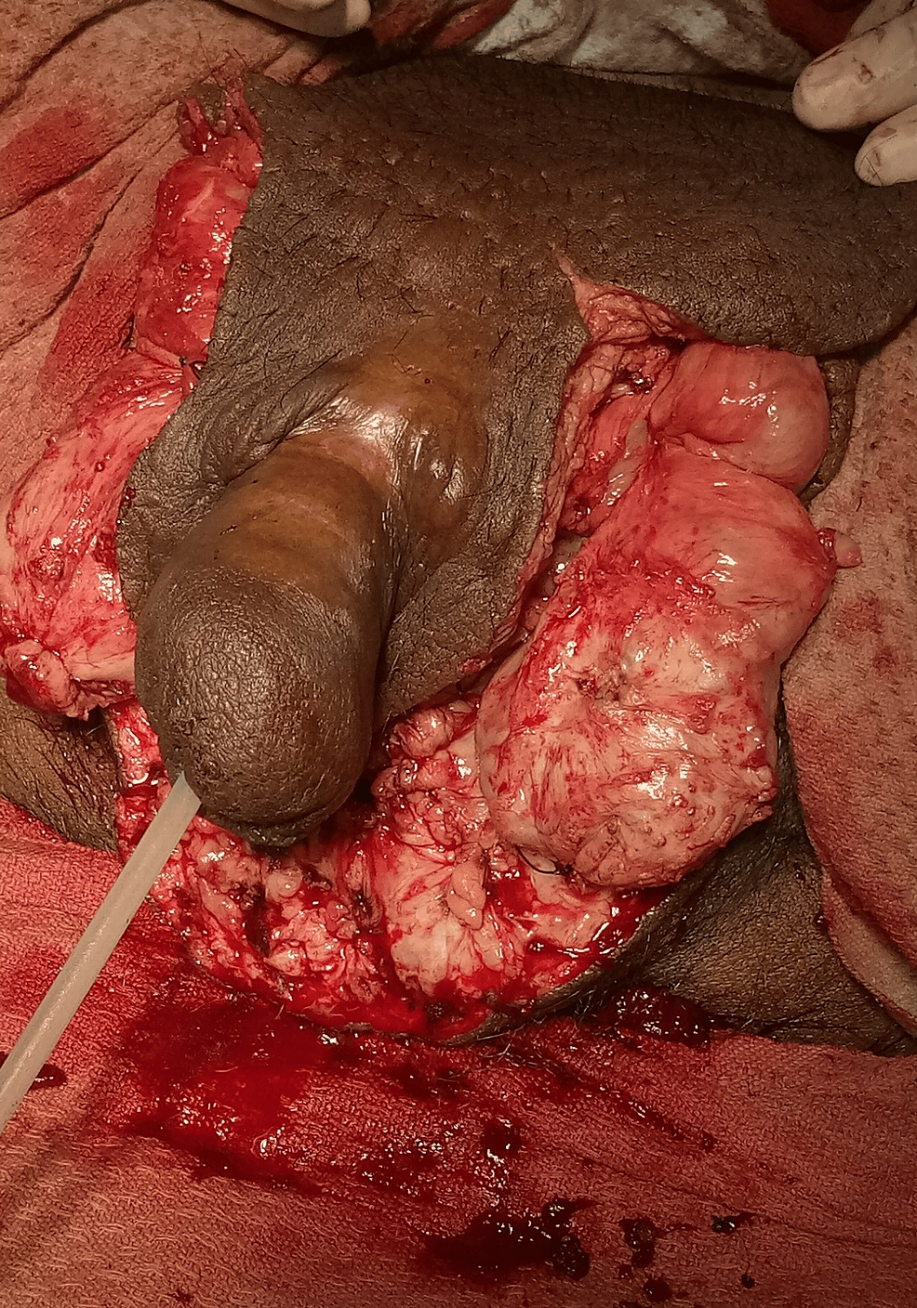
Intraoperatively, after the resection of all lymphoedematous tissue.

The patient was planned for surgery after clearance from the pre-anaesthetic clinic. Pre-operative urethral catheterisation was done after localising the urethral meatus. Two separate incisions were made in the bilateral inguinal region. Penis was delineated circumferentially near its root from the scrotal skin using the electrocautery method, and scrotal skin flaps were raised by extending the incision horizontally outwards. After careful dissection of scrotal tissues, bilateral dilated tortuous vessels were ligated. Bilateral testes were examined and found to be healthy. Fibrotic tissues were resected, and the remaining scrotal skin flaps were closed in a Y-shaped manner. The patient underwent debulking surgery where most of the fibrosed scrotal skin and underlying soft tissues were removed. The total duration of surgery was about 2 hours and 30 minutes, and the weight of scrotal tissue measured after excision was about 35 kg (Figures 1-2). The post-operative period was uneventful. The patient was kept on intravenous broad-spectrum antibiotics (piperacillin-tazobactam and clindamycin) post-operatively and was discharged on oral amoxicillin. During follow-up visits, the flap was healthy.

Case 2

**Figure 3:**
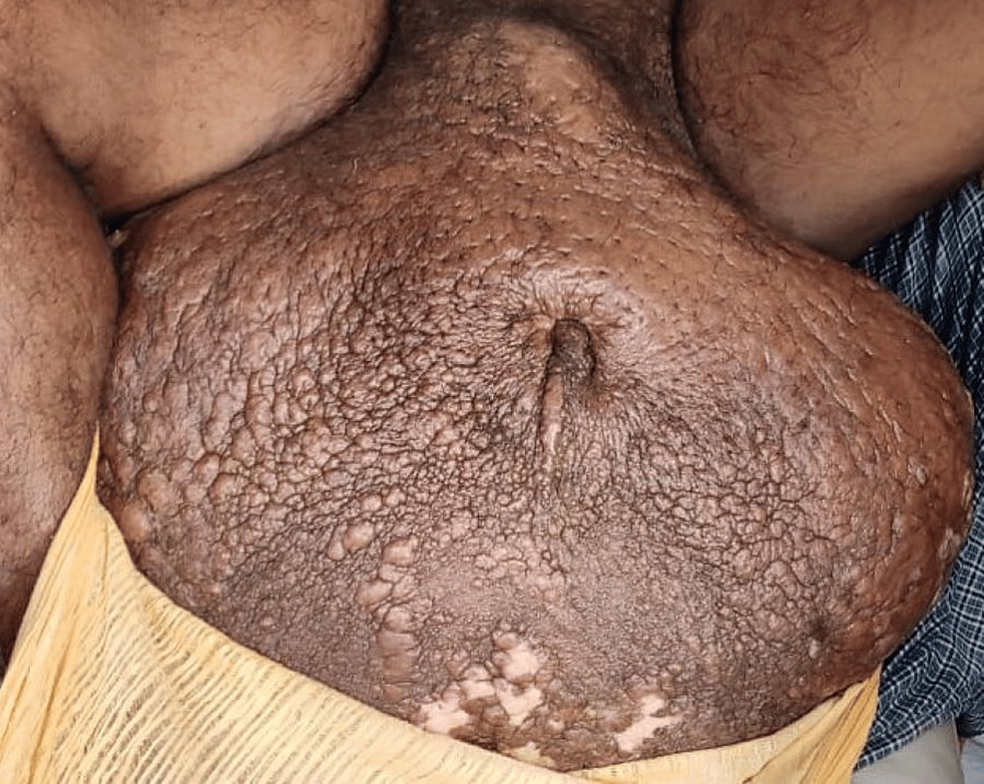
Pre-operative grossly enlarged scrotal with fissuring and ulceration over the scrotal skin.

**Figure 4:**
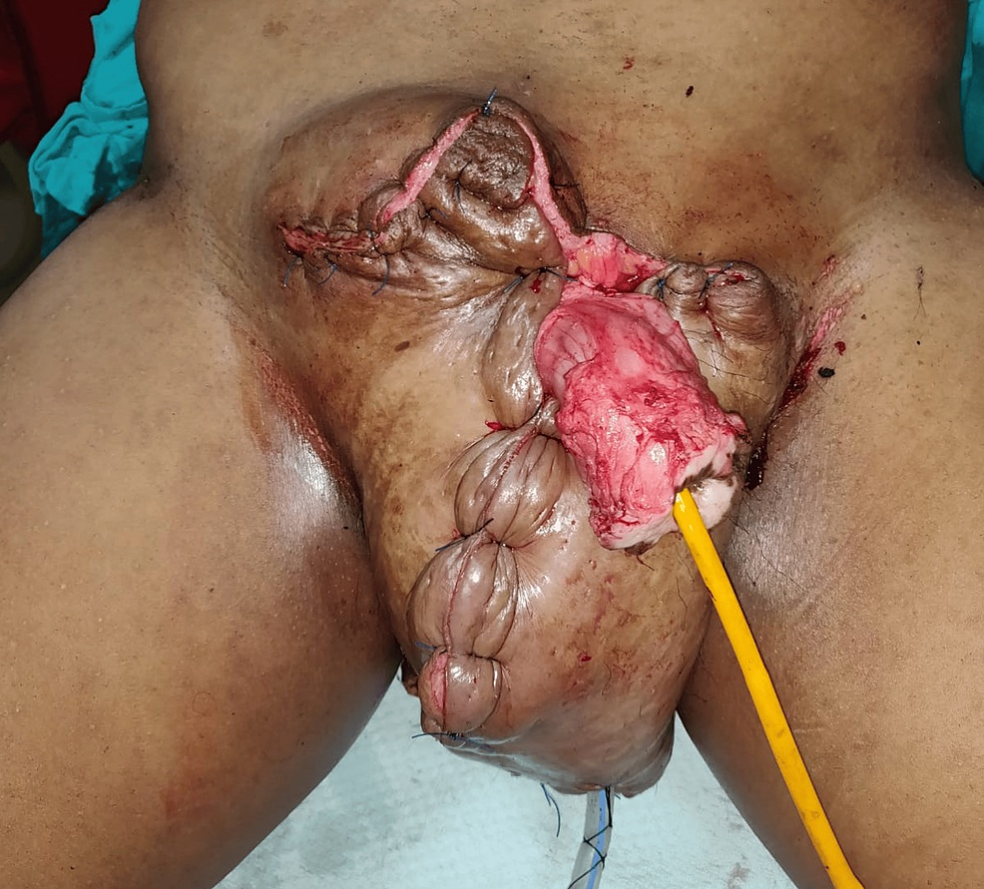
Intraoperatively, after debulking and reconstruction of the scrotum.

**Figure 5:**
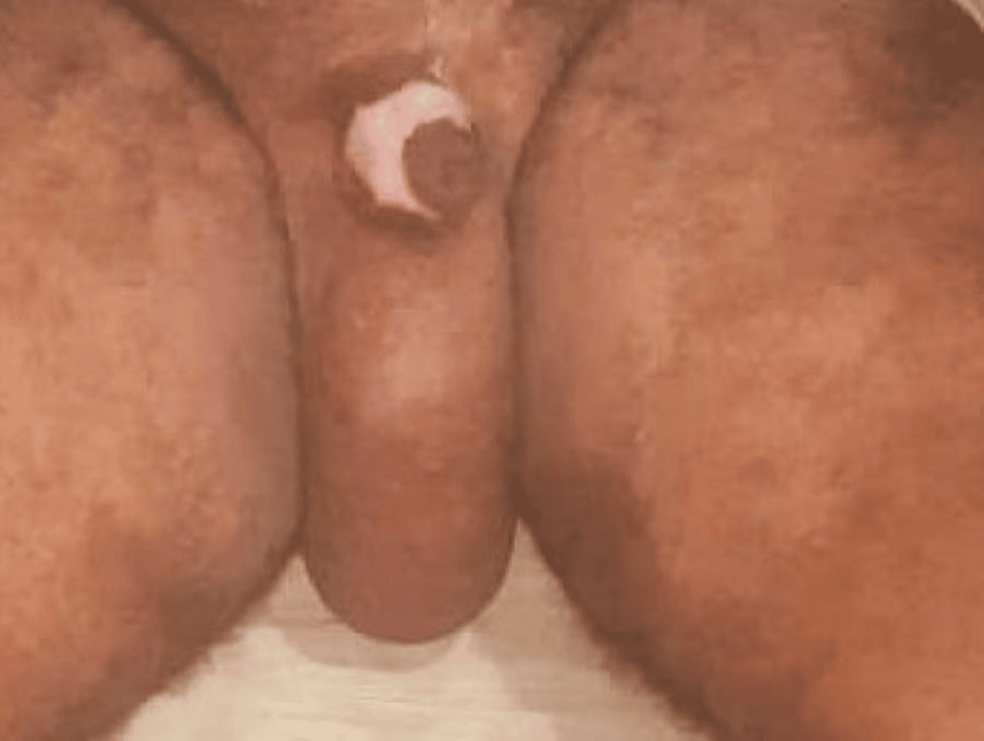
Post-operative status of the scrotal skin, three months after surgery.

A 45-year-old gentleman who was a farmer by profession presented with an enlarged scrotum, which hindered his ability to walk due to the weight of the scrotum. The patient started developing the condition around seven years back when he was diagnosed with hydrocoele. He was operated six years back in a private hospital for hydrocoele. The condition recurred after three months post-surgery and eventually progressed to the present size, with skin changes over the scrotum. Physical examination revealed a scrotal size of 65 cm x 60 cm x 52 cm, with multiple deep pits over the scrotal skin and a large infected ulcer over the dependent aspect of the scrotum. Penis was buried in scrotal tissue; however, pre-operative Foley catheterisation helped in localising penile tissue intraoperatively. Pre-operatively, the infection was controlled using antibiotics and regular dressings. Once the scrotal skin was free of infection, the patient was posted for debulking surgery after seeking clearance from the pre-anaesthetic clinic. The scrotal skin flap was fashioned similarly as in the previous case, and edematous tissues were excised. The penile skin was denuded due to its involvement with lymphoedema. Care was taken to include the ulcer in the excised tissue. The duration of the procedure was about 3 hours and 30 minutes, and the excised scrotal tissue weighed 32 kg. The patient was shifted to the intensive care unit during the immediate post-operative period for post-operative monitoring and was discharged eventually after 14 days. Sutures over scrotal skin were removed on 10 days postoperatively. The patient required revision surgery for coverage of penile tissue after three months (Figures 3-5).

On post-operative day 3, there was evidence of serous discharge from the stitch line, which was managed by daily dressing. Post-operatively, piperacillin-tazobactam and linezolid were administered intravenously. Once the infection was controlled, the patient was discharged on oral amoxicillin. During follow-up visits, the flap was found to be healthy.

Case 3

**Figure 6:**
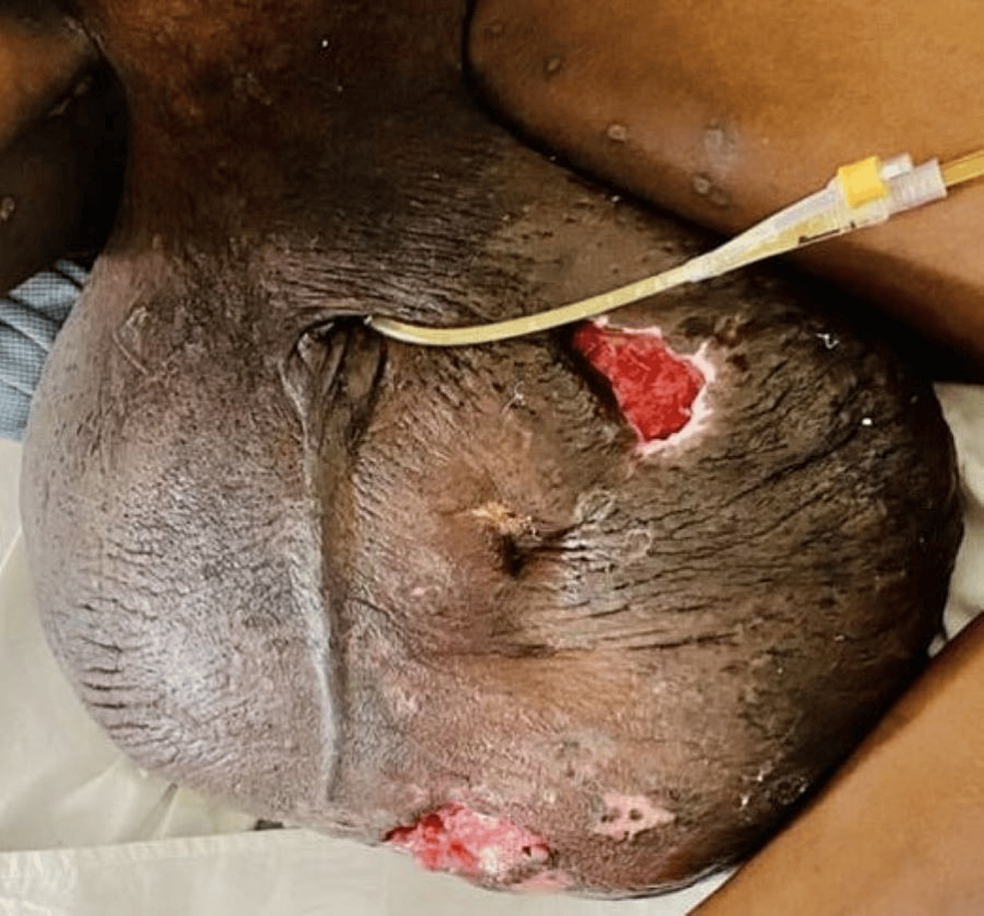
Pre-operative status of the scrotal skin with multiple healing ulcers over the scrotum.

**Figure 7:**
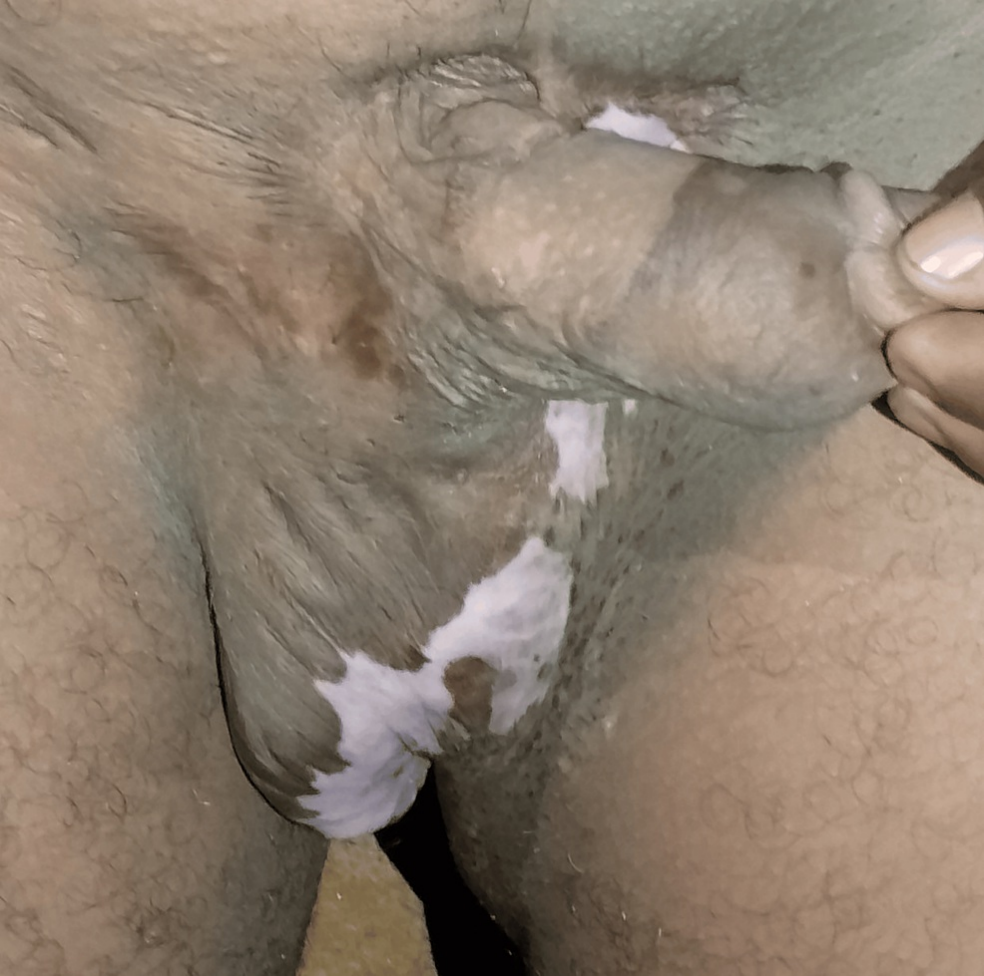
Post-operative status of the scrotum, three months after debulking and reconstructive surgery.

A 22-year-old man presented with a similar history of 10 years along with the involvement of penile tissue. There was no history of any surgical intervention or radiation exposure. The testis was found to be atrophic on high-resolution ultrasonography of the inguinoscrotal region, and the patient was accordingly counselled for orchidectomy. Once the patient was posted for surgical debulking, urethral catheterisation was done pre-operatively. The scrotal skin flap was fashioned similarly, and debulking was performed. As the penile skin was involved, it was excised, and grafting of the penile skin was performed approximately six months after the initial surgery. The duration of the procedure was about 3 hours, and excised scrotal tissue weighed 25 kg. Post-operatively, the patient experienced a minor wound infection on postoperative day (POD) 5, which was managed through regular dressings and antibiotic therapy. The patient was given intravenous piperacillin and clindamycin for seven days, followed by oral amoxicillin (Figures 6-7). During follow-up outpatient visits, the flap was found to be healthy.

Patients presented to the outpatient clinic of the Department of General Surgery, KGMU. The demographic profile of the patients was recorded, including a history of comorbidities such as diabetes, hypertension, cardiac illness, and past medical and surgical history. Clinical examination was performed, and scrotal size, skin changes (fibrosis and papillae), and ulcerations were noted. The weights of the patients before and after surgery were recorded. The diagnosis of filariasis was established based on clinical findings and the endemicity of the disease.

The patients were followed for three months post-surgery. Incidences of surgical site infection were noted on days 1, 3, 7 and 30 after the surgery. Any evidence of wound dehiscence and seroma was noted during follow-up visits. Any evidence of recurrence was recorded on days 30 and at three months. Cosmetic results were assessed after three months.

## Discussion

Genital lymphoedema is a relatively rare entity, which causes severe grades of disability in patients, especially with giant variants. Lymphoedema of the penis and scrotum is due to abnormal accumulation of lymphatic fluid in the subcutaneous tissue of the penis and the scrotum. Lymphatic filariasis is the leading cause of such presentation and can be identified and treated in the early stages [6]. In endemic areas, patients often present late during the course of the disease owing to social stigmata and poor financial background. They often tend to seek help from local pharmacists, herbalists, hakims and sorcerers, or end up self-treating. Lymphatic filariasis obstructs proximal lymphatic channels, resulting in dilatation of the distal lymphatic channels. Chronic lymphocytosis leads to the accumulation of interstitial fluid, protein growth factors and other active peptide moieties, glycosaminoglycans and particulate matter, including bacteria [7]. This results in an increase in collagen production by fibroblast, translocation of inflammatory cells (predominantly macrophages and lymphocytes) and keratinocyte activation. Initial stages may present as hydrocoele alone without any other complication or skin changes. Gradually, fibrosis sets in, and the skin becomes coarse with a pitted appearance and massive enlargement of the genital skin. Patients may present with recurrent lymphangitis, cellulitis, ulcerations, lymphorrhoea and vesiculations of scrotal skin [8].

Diagnosis is made from the patient's history and examination. Supportive evidence can be obtained by imaging studies such as ultrasonography. Card tests for filarial antigens can be performed; however, negative tests cannot rule out the disease. While radionuclide lymphoscintigraphy is the standard imaging study for evaluation, it could not be performed on our patients due to financial limitations.

Early recognition is essential for treating the condition. The disease is curable in the acute stage. For initial grades of lymphoedema, medical therapy can be initiated to halt the disease progression. The role of conservative therapy is limited to early stages, and the effects are usually temporary and require long-term follow-up.

Lymphaticovenular anastomosis [9] can be performed in cases where fibrosis is not evident; however, it demands surgical expertise as well as has limited success rates. In conditions where fibrosis has set in, debulking surgery (also called reduction scrotoplasty) is the standard of care [10] and yields acceptable post-operative results.

## Conclusions

Giant scrotal lymphoedema is a distressing condition causing both physical and psychosocial distress to the patient. In this case series, it was noted that the patients presented later in the course of the disease. The primary reason for this delay can be attributed to the physical disabilities of the patients and the associated social stigma. In all the cases, resection of fibrosed scrotal tissue was done along with the reconstruction of the external genitalia and had acceptable cosmetic results that proved life-changing for these patients. Excisional surgery with reconstruction is the mainstay of the treatment. The reconstruction of massive scrotal lymphoedema was achievable with the scrotal sizes weighing 35, 32 and 25 kg in cases 1, 2 and 3, respectively. Complete pre-operative clinical evaluation is necessary to understand the extent of the disease. Pre-operative urethral catheterisation helped in the localization of penile tissue intraoperatively and provided a guide for limits of resection. Post-operative wound management as well as antibiotic therapy played an essential role in the recovery of the patients. Close follow-up is essential for evaluating flaps and assessing the effectiveness of the procedure.

## References

[1] Denzinger S, Watzlawek E, Burger M, Wieland WF, Otto W (2007). Giant scrotal elephantiasis of inflammatory etiology: a case report. J Med Case Rep.

[2] McDougal WS (2003). Lymphedema of the external genitalia. J Urol.

[3] Kuepper D (2005). Giant scrotal elephantiasis. Urology.

[4] Brown WL, Woods JE (1977). Lymphedema of the penis. Plast Reconstr Surg.

[5] Bulkley GJ (1962). Scrotal and penile lymphedema. J Urol.

[6] Thejeswi P, Prabhu S, Augustine AJ, Ram S (2012). Giant scrotal lymphoedema - a case report. Int J Surg Case Rep.

[7] Apesos J, Anigian G (1991). Reconstruction of penile and scrotal lymphedema. Ann Plast Surg.

[8] Rupinderjit K (2011). Giant scrotal elephantiasis of idiopathic aetiology. JARBS.

[9] Ferdinand NW, Peter BK (2018). Bilateral scrotal flaps: a novel idea in the management of massive scrotal lymphoedema. J Surg.

[10] Parmar HD (2013). The surgical approach in huge scrotal lymphoedema. Int J Med Sci Public Health.

